# A red/blue bicolor lateral flow immunoassay for simultaneous detection of two enteroviruses based on duplex RT-LAMP

**DOI:** 10.3389/fmicb.2026.1787049

**Published:** 2026-03-18

**Authors:** Ting Zhou, Alang Zhang, Hengxuan Zhou, Zhihua Xu, Xinyu Cheng, Chenxi Guo, Yuankun Wang, Jiahui Wu, Feng Shi, Yishan Ding

**Affiliations:** 1 College of Life Sciences, Shihezi University, Xinjiang, Shihezi, China; 2 Traditional Chinese Medicine Hospital of Xinjiang Production and Construction Corps, Kuitun, China

**Keywords:** acute gastroenteritis, human adenovirus, lateral flow immunoassay (LFIA), loop-mediated isothermalamplification (LAMP), norovirus, point-of-care diagnosis, visual detection

## Abstract

**Introduction:**

Norovirus and human adenovirus are the main pathogens causing acute gastroenteritis (AGE) worldwide. Their highly contagious nature, significant disease burden, similar symptoms, and frequent co-infection necessitate a differential diagnostic tool to guide epidemic control and support antimicrobial stewardship by rapidly ruling out bacterial etiologies.

**Methods:**

This study developed an integrated dual reverse transcription loop-mediated isothermal amplification (RT-LAMP) and dual-color lateral flow immunoassay (LFIA) technology for rapid, sensitive, and visual synchronous detection of norovirus genogroup II (NoV GII) and human adenovirus F41 (HAdV F41) in human feces. LAMP primers for the two viruses were labeled with FAM/biotin and TAMRA/biotin, respectively. Combined with a dual-color gold nanomaterial signaling system (red AuNPs for test lines, blue AuNFs for control line), the assay allows for intuitive visual interpretation.

**Results:**

The method demonstrated good sensitivity and specificity, with detection limits of 5.8 copies/μL for NoV GII and 63 copies/μL for HAdV F41, and no cross-reactivity with common non-target pathogens. The entire reaction process requires only a constant temperature of 65°C and can be completed within 45 min.

**Discussion:**

The clear dual-color interpretation system, user-friendly operation, and excellent performance make it a highly promising tool for first-line screening and point-of-care diagnosis of AGE.

## 1 Introduction

Acute gastroenteritis (AGE) is a rapidly developing infectious intestinal disease characterized primarily by increased bowel movements and loose or watery stools, often accompanied by nausea, vomiting, fever, or abdominal pain, and unrelated to pre-existing chronic conditions. As a major cause of illness and death among children globally, AGE can be caused by various pathogens, with enteroviruses being the primary causative agents (Alves et al., 2024).

Norovirus (NoV) is now recognized as the leading global cause of AGE, resulting in over 600 million cases and approximately 200,000 deaths annually, with particularly severe impacts on young children, the elderly, and immunocompromised individuals. NoV is a non-enveloped, positive-strand RNA virus currently classified into at least 10 genotypes, with genotype II (GII) being the predominant one (Carlson et al., 2024). The GII.4 strain not only causes the most severe cases and large-scale outbreaks but also exhibits significant temporal variation and frequent recombination events. Epidemiological surveillance indicates that NoV detection rates in pediatric AGE cases range from 12 to 20%, exceeding 40% in hospitalized cohorts. Beyond the recurrent dominance of the GII.4 Sydney lineage, multiple genotypes including GII.2, GII.3, GII.6, GII.13, and GII.17 coexist widely (Lu et al., 2019; Li et al., 2021; Avellaneda et al., 2025). The extremely low infectious dose, rapid fecal-oral transmission route, and strong environmental stability make outbreaks difficult to control. Hospitals, daycare centers, and long-term care facilities are thus high-risk settings for outbreaks (Afework et al., 2022; Winder et al., 2022; Zhang et al., 2024; Wang et al., 2025).

Human adenovirus F40/41 (HAdV-F40/41) represents another major AGE pathogen, causing an estimated 50,000 deaths annually among children under five years old. Its mortality burden ranks third to rotavirus and Shigella (Do Nascimento et al., 2022; Li et al., 2025). HAdV was detected in 4–25% of pediatric AGE samples, with F41 as the predominant genotype. This serotype exhibits high environmental stability, with persistent viral shedding and prolonged viability on surfaces.

NoV and HAdV-F41 are frequently co-detected with other enteroviruses (e.g., rotavirus), and both cause highly similar clinical presentations—acute watery diarrhea, vomiting, and fever—making symptomatic differentiation challenging (Do Nascimento et al., 2022; Ofosu-Appiah et al., 2025). Studies have found that viral loads of HAdV are significantly lower in mixed infections compared to single infections, suggesting its pathogenic role may be masked. This further underscores the necessity for accurate pathogen identification (Hassan et al., 2019; Kinzler et al., 2025). In clinical management, there are currently no specific antiviral drugs targeting NoV or HAdV. Treatment primarily relies on symptomatic supportive measures, including fluid replacement and maintenance of electrolyte balance. However, establishing a definitive pathogen diagnosis holds significant value: it effectively avoids unnecessary antibiotic use while aiding in more precise assessment of dehydration risk, predicting disease progression, and formulating individualized supportive treatment strategies (Arbanas et al., 2025).

Currently, detection methods for NoV and HAdV fall into two main categories, each with inherent limitations. The biggest advantage of the molecular detection method, represented by qPCR, is its high sensitivity, good specificity, and accurate typing and quantification (Li et al., 2022; Sánchez-Sánchez et al., 2023). It is the primary choice for diagnosis and epidemic tracing, but it relies on complex instruments, skilled personnel, professional laboratories, and is time-consuming, making it unsuitable for point-of-care testing (POCT) (Coudray-Meunier et al., 2016; Malik et al., 2019). The core advantage of the rapid antigen detection method represented by rapid antigen methods (ELISA, lateral flow/ICT) is that it is relatively simple and fast to operate, does not need special equipment, and is suitable for rapid clinical screening and initial judgment of on-site epidemic situation, but the biggest disadvantage is that the sensitivity is significantly lower than that of molecular methods, especially when the viral load is low or the disease is late, it is easy to miss detection (Richards et al., 2003; Geginat et al., 2012; Ushijima et al., 2024). Isothermal amplification technology, such as loop-mediated isothermal amplification (LAMP), has become a promising alternative method due to its simple and rapid reaction conditions (Bhadra et al., 2020; Garg et al., 2022; Yang et al., 2024). However, traditional Lamp detection methods usually rely on turbidity, pH, or non-specific dyes. These methods can only report “amplification” but cannot determine the target of the amplification. For example, when multiple targets cannot be distinguished in a test tube or multiple primers are used, the risk of non-specific amplification and false positives is higher (Fan et al., 2022; Crego-Vicente et al., 2024). Therefore, in recent years, there have been some new detection methods based on LAMP that can simultaneously detect multiple viruses, improving diagnostic efficiency (Gadkar et al., 2018; Das et al., 2022; Nak-on et al., 2025; Zhuang et al., 2025).

To bridge the critical gap between laboratory-level accuracy and the simplicity of field deployment, we developed a detection method using RT-LAMP combined with two-color LFIA for the simultaneous visual detection of Nov GII and HAdV-F41 viruses. Here, we combine the high sensitivity of multiplex nucleic acid amplification with the intuitive visual readout of LFIA. The core innovations include: (1) designing and modifying specific primers for the targets of the two viruses to establish a dual RT-LAMP reaction system; (2) development of a novel dual-color gold nanomaterial signal system using red gold nanoparticles (AuNPs) and blue flower-like gold nanoparticles (AuNFs) with distinct optical properties, which minimizes the risk of visual misinterpretation and is particularly suitable for on-site rapid screening by non-specialists; (3) incorporation of integrated contamination prevention strategies, adopting the combination of dUTP/UDG inhibitors with mineral oil coatings has effectively controlled the problem of cross-contamination. We comprehensively evaluated the analytical performance of the assay, including sensitivity, specificity, and stability, and verified its clinical utility using stool specimens. The aim of this study is to develop a dual functional LAMP-LFIA platform for the simple, rapid, highly sensitive, and specific detection of two major enteroviruses, thereby promoting improved epidemiological surveillance in resource limited environments and providing supportive treatment strategies to avoid unnecessary antibiotic use.

## 2 Materials and methods

### 2.1 Reagents and apparatus

Bst 2.0 WarmStart^®^ DNA Polymerase, MgSO_4_, WarmStart^®^ RTx Reverse Transcriptase, 10 × isothermal amplification buffer, and LAMP fluorescent dyes were purchased from New England Biolabs (Ipswich, MA, United States). Thermally unstable uracil-DNA glycosylase (UDG) and dNTPs were obtained from Vazyme Biotech Co., Ltd. (Nanjing, China). Anti-fluorescein isothiocyanate (FITC) rabbit polyclonal antibody was provided by Shanghai Biological Engineering Co., Ltd., Chicken yolk immunoglobulin (IgY) and goat anti-chicken IgY antibody were purchased from Biodragon Technology Co., Ltd. (Suzhou, China). Rhodamine-marked antibodies and streptavidin were purchased from Bio Basic Inc. (Markham, ON, Canada). Cellulose nitrate (NC) membranes, sample pads, and combination pads were acquired from Millipore Sigma (Darmstadt, Germany). All LAMP primers were supplied by Shanghai Sangon Biotechnology. The remaining chemical reagents were obtained from Shanghai McLean Biochemical Technology Co., Ltd. Nucleic acid standard products of NoV GII, rotavirus A (RVA), enterovirus A71 (EVA71), Coxsackie virus A16 (CVA16) and HAdV F41 were purchased from BeNa Culture Collection. Amplification was performed using a Bioer LineGene 9660 Plus qPCR instrument (Hangzhou Bioer Technology Co., Ltd.)

### 2.2 Design of LAMP primers

For LAMP primer design, NoV GII selected the VP1 target gene, GenBank accession number X86557.1; The target of HAdV-F41 is the Hexon gene, with GenBank accession number PP921917.1 (Fukuda et al., 2006; Yoda et al., 2007; Liang et al., 2025). The genomic sequences of these specific regions were retrieved, and their conservation was verified through alignment with circulating strains before design. Then, specific LAMP primer sets for each virus were designed using the online software Primer Explorer V5 (Eiken Chemical Co., Ltd., Japan). Each set included two outer primers (F3 and B3), two inner primers (Forward Inner Primer, FIP; Backward Inner Primer, BIP), and loop primers (LF and LB) to accelerate the amplification reaction (For NoV GII, an LB primer could not be optimally designed within the conserved VP1 region; therefore, only the LF loop primer was included. This configuration was validated to provide sufficient amplification efficiency and specificity.). All designed primers were checked for specificity through *in silico* analysis against the NCBI nucleotide database. To enable subsequent capture and detection on the dual-color lateral flow immuno-chromatographic strip, the primer sets were strategically labeled. The FIP primers for both viruses were modified at their 5′ end with biotin. The BIP primers were differentially labeled at the 5′ end with distinct haptens: the NoV GII BIP was conjugated with 6-carboxyfluorescein (FAM), and the HAdV-F41 BIP was conjugated with tetramethyl-rhodamine (TAMRA). These haptens correspond to antibodies immobilized on the test lines of the strip. All oligonucleotides were synthesized and purified by HPLC by Sangon Biotechnology Co., Ltd. (Shanghai, China). The complete nucleotide sequences of all primers are listed in Table 1.

**TABLE 1:** The specific primers designed for NoV GII and HAdV-F41 detection.

Virus	Target gene	Primer	Sequence 5′–3′	Modifi-cation
NoV GII	VP1	F3 B3 FIP BIP LF LB^1^	GGCTCCCAGC TTTGTGAAT CGCTCCACA GTATCTCACCT CGGGCTCCAGAGC CATAACCTTGA CGCCAACCCA TCTGAT GTGGCGGGCC AACAAAACGTAAC TGTGAACTCTCC ACCAGG ATTGACCTCTGGG ACGAGG Not designed	FIP: 5′-Biotin BIP: 5′-FAM
HAdV-F41	Hexon	F3 B3 FIP BIP LF LB	ACCCCGCCA ACTATCCAT TCATCCATGG GGTCCACC CGCCACATGGTACG ATCCAAAACCCCC TTATTGGTCAGACG TATGTCTATGGGG GCCCTGACC TCAAAAG TCATGTCGAGCGC AGTCAGGCT TGGTACGGC GACCTGGGG CAAAACATGC	FIP: 5′-Biotin BIP: 5′-TAMRA

^1^For NoV GII, no suitable LB primer could be designed within the target region; preliminary experiments confirmed that amplification with only LF primer was efficient and specific.

### 2.3 Establishment of the duplex RT-LAMP reaction system

A 25-μL reaction mixture was assembled on ice, containing: 2.5 μ L of 10 × isothermal amplification buffer, 6 mM MgSO4,3.5 μ L of a dNTP mixture (1.4 mM final concentration, with dUTP fully substituting dTTP), 1 μ L of an enzyme mix containing 8 U of Bst 2.0 DNA polymerase and Warm Start RTx reverse transcriptase, 2.5 μ L of a 10 × primer mixture(final concentrations for each virus: 0.8 μ M FIP and 0.8 μ M BIP for NoV GII; 1.6 μ M FIP and 1.6 μ M BIP for HAdV-F41. The concentrations were 0.2 μM for F3 and B3, and 0.4 μM for LF and LB and 2 μL of template nucleic acid. To allow real-time monitoring of amplification kinetics without affecting endpoint lateral flow detection, 1 × commercial fluorescent dye (e.g., NEB Colorimetric or SYBR Green I) was added. The final volume was adjusted with nuclease-free water. The reaction was incubated at 65°C for 45 min in a qPCR instrument or a thermal block. An integrated anti-contamination strategy was incorporated into the workflow. After LAMP incubation, to reduce cross-contamination and avoid false positive results on subsequent test strips. A UDG-based system was employed to prevent carryover contamination. In the reaction premix, dTTP was partially substituted with dUTP, resulting in amplification products containing uracil (U). These uracil-containing amplicons are susceptible to degradation by UDG, thereby preventing carryover contamination in subsequent reactions. Before the new reaction begins, the heat-sensitive UDG enzyme premixed in the system is activated at low temperature (37°C, 5 min). It can specifically identify and degrade any “fragile” products that may contaminate the system in the previous experiment, thus eliminating the possibility of them acting as templates. When the reaction temperature rises to the LAMP amplification temperature (65°C), the UDG enzyme is quickly and completely inactivated, thus ensuring that the template DNA to be tested in the reaction is not degraded and the normal progress of the amplification. In addition, after all liquid components were added to the reaction tube, the mixture was overlaid with 20 μL of sterile mineral oil to minimize aerosol formation and cross-contamination.

### 2.4 Optimization of the duplex RT-LAMP assay conditions

Through experimental verification, adjusting the primer ratio of different targets can achieve stable single-tube reaction and specific simultaneous output of multiple LAMP amplification products. Therefore, we evaluated the amplification efficiency of NoV GII and HAdV F41 with different primer ratios (1:1, 1:2, 1:3, 2:3, 3:2, 2:1) by monitoring the total amplification signal (Liu et al., 2025). Validate the dual RT-LAMP system using nucleic acid standards (10^3^copies/μL), and set each primer ratio reaction tube to contain two virus templates (1 μL each of 10^3^ copies/μL stock solutions mixed, total template 2 μL, ensuring an initial quantity of 10^3^ copies for each target) as the experimental group; And set two viruses (single template, single primer) as controls. The amplification kinetics of each proportion were evaluated by the real-time fluorescence amplification curve, and finally determined that 3:2 is the best ratio of two viral primers. Then, agarose gel electrophoresis and LFIA were used to detect the amplification products under the condition of a 3:2 primer ratio to prove the feasibility of this method. With the primer ratio fixed at 3:2, amplification was evaluated at 57, 60, 63, 65, and 67°C to determine the optimal reaction temperature.

### 2.5 Synthesis of gold nanomaterial-labeled conjugates

Red colloidal AuNPs with an average diameter of approximately 40 nm were synthesized as the first colorimetric label via the classical citrate reduction method (Memon et al., 2022). Briefly, 5mL of the freshly prepared AuNP solution was adjusted to pH 8.0 using 0.2M K_2_CO_3_. Streptavidin (SA) solution (70μL, 1mg/mL) was then added dropwise under gentle stirring, and the mixture was incubated overnight at 4°C to allow SA conjugation.

AuNFs were synthesized using HEPES as a reducing agent and particle stabilizer, serving as a second chromogenic label (Fang et al., 2021). A 5mL aliquot of the synthesized AuNF dispersion was adjusted to pH 7.5 with 0.2M K2CO3, followed by the slow dropwise addition of 50μL of IgY solution (1mg/mL) under continuous stirring. The mixture was left to stand overnight at 4°C to form IgY-AuNF conjugates.

To block non-specific binding sites, the SA-conjugated AuNPs (SA-AuNPs) and IgY-conjugated AuNFs (IgY-AuNFs) were each mixed with 0.5mL of 10% (w/v) bovine serum albumin (BSA) solution and incubated at room temperature for 1h. Subsequently, the conjugates were purified by centrifugation at 10,000 × g and 4°C for 30min. The supernatant was discarded, and the resulting pellets were resuspended in 250μL of storage buffer (e.g., PBS with 1% BSA or appropriate suspension buffer). The final conjugates were stored at 4°C until use.

The morphology of AuNPs and AuNFs was characterized by transmission electron microscopy (TEM), and their optical properties were analyzed by ultraviolet–visible (UV-Vis) spectroscopy. Detailed synthesis protocols, including reagent concentrations and reaction conditions, are provided in Supplementary Material S1.

### 2.6 Construction of the duplex LFIA strip

The bicolor detection conjugates—streptavidin-conjugated colloidal gold nanoparticles (SA-AuNPs) and IgY-conjugated gold nanoflowers (IgY-AuNFs)—were mixed at a 1:1 (v/v) ratio in a dispensing buffer. This mixture was uniformly sprayed onto the pretreated conjugate pad at a controlled rate of 30 μL/cm and then dried at 37°C for 12 h. The test lines were dispensed onto the nitrocellulose membrane at a rate of 1μL/mm: the T1 line was coated with anti-FITC monoclonal antibody to specifically capture FAM-labeled NoV GII amplicons, the T2 line with anti-TAMRA monoclonal antibody to capture TAMRA-labeled HAdV-F41 amplicons, and the control (C) line with goat anti-chicken IgY to bind residual IgY-conjugated gold nanoflowers (IgY–AuNFs), thereby confirming strip integrity and proper fluid flow. Following dispensing, the membrane was dried at 37°C for 12 h. Finally, the assembled card was precisely cut into individual test strips 3 mm wide. The dual LFIA is constructed by sequentially laminating a nitrocellulose (NC) membrane, a binding pad, a sample pad, and an absorbent pad onto a polyvinyl chloride (PVC) backing plate, with a 2 mm overlap between adjacent components. Both the sample pad and the binding pad are pretreated with a blocking buffer (e.g., PBS containing 1% BSA, 0.5% Tween-20, and 0.05% Sodium Azide, pH 7.4) and dried before assembly (Mirica et al., 2022). The finished strips were packaged in sealed aluminum foil bags containing desiccant and stored at room temperature in a dry, dark environment until use.

### 2.7 Optimization of the bicolor lateral flow immunoassay strip

After assembling the test strips, key parameter ratios were optimized to enhance the sensitivity, specificity, and signal clarity of the dual-color, dual-side-flow immunoassay. The ideal working concentrations for SA with colloidal AuNPs and IgY with AuNFs were determined via standard NaCl-induced flocculation assays (Geoghegan, 1988; Mirón-Mérida et al., 2021). Subsequently, the volume mixing ratio of SA-AuNP and IgY-AuNFs complexes formed on the conjugate pad was optimized by testing ratios of 1:2, 1:1, 2:1, and 3:1 (volume ratio) to achieve a balance in color intensity between the two test lines. Finally, the concentrations of the capture reagents immobilized on the nitrocellulose membrane were independently fine-tuned: the goat anti-chicken IgY for the control (C) line was tested at 0.05, 0.1, 0.25, 0.5, 0.75, 1.0, 1.5, and 2.0 mg/mL; the anti-FAM monoclonal antibody for the T1 line (specific for NoV GII amplicons) at 0.2, 0.4, 0.6, 0.8, and 1.0 mg/mL; and the anti-TAMRA monoclonal antibody for the T2 line (specific for HAdV-F41 amplicons) at 0.1, 0.25, 0.5, 1.0, 1.5, and 2.0 mg/mL. All optimization experiments were performed using LAMP amplicons of each target at concentrations near the preliminary limit of detection as the challenge samples. The final parameter set was selected based on the criteria of yielding the strongest specific signal intensity, the sharpest band morphology, and the cleanest background for both targets simultaneously in the duplex format.

### 2.8 Analytical performance evaluation of the duplex LAMP-LFIA assay

#### 2.8.1 Determination of analytical sensitivity

The analytical sensitivity of the optimized duplex LAMP-LFIA assay was evaluated using serial 10-fold dilutions of quantitative synthetic standards. The NoV GII RNA standard was diluted from an initial concentration of 5.8 × 10^4^ copies/μL down to 5.8 × 10^–2^ copies/μL, and the HAdV-F41 DNA standard from 6.3 × 10^4^copies/μL down to 6.3 × 10^–2^ copies/μL. For each dilution, six independent replicate LAMP reactions were performed under the optimized conditions (65°C for 45 min). Nuclease-free water served as the negative control in each run. Following amplification, a 1 μL aliquot was diluted in 100 μL of dedicated running buffer and applied to the LFIA strip for visual readout. The assay was repeated three times for each concentration of RNA/DNA.

#### 2.8.2 Assessment of analytical specificity

The analytical specificity of the assay was assessed against a panel of non-target viral pathogens commonly associated with gastroenteritis or clinically similar presentations. The panel included quantitative standards for Rotavirus A (RV-A), Coxsackievirus A16 (CV-A16), and Enterovirus A71 (EV-A71). Each non-target pathogen was tested individually at a high concentration of approximately 10^4^ copies/μL. Additionally, a mixture containing all three non-target viruses was prepared and tested to evaluate potential cross-reactivity or interference in a complex sample matrix. All specificity tests were performed in triplicate under the standard assay conditions. A test was considered specific if the LFIA strips showed no signal development at either the NoV GII-specific (T1) or HAdV-F41-specific (T2) test lines, while the control (C) line developed normally, confirming the valid function of the strip.

#### 2.8.3 Assessment of strip stability

The storage stability of the assembled LFIA test strips was evaluated using an accelerated aging protocol (Kaur et al., 2021). Freshly prepared strips were stored in sealed aluminum foil bags containing desiccant at 37°C for 3, 7, and 15 days. At each time point, strips were removed and tested using NoV GII and HAdV-F41 nucleic acid standards at concentrations near the limit of detection (approximately 10^1^ copies/μL for NoV GII and 10^2^ copies/μL for HAdV-F41, corresponding to approximately 1.6–1.7 × LOD). All tests were performed in triplicate, and signal intensity was compared visually with freshly prepared strips.

### 2.9 Clinical validation with real stool samples

For clinical validation, 33 residual fecal samples were retrospectively collected from the Traditional Chinese Medicine Hospital of the Xinjiang Production and Construction Corps, Kuitun, China, between April and November 2025. Total nucleic acids were extracted from 10% (w/v) fecal suspensions using the FastPure^®^ Viral DNA/RNA Mini Kit Pro (Vazyme Biotech Co., Ltd.) according to the manufacturer’s instructions. All extracts underwent initial identification using the Guangzhou Daan Gene Company RT-qPCR kit as the reference standard. Based on RT-qPCR results, the samples comprised 9 NoV-positive, 10 HAdV-positive, 8 NoV and HAdV co-infection-positive, and 6 negative samples. Subsequently, a fully optimized dual RT-LAMP-LFIA detection protocol was employed to test each extracted nucleic acid sample in a blinded manner (operators unaware of RT-qPCR results). Amplification conditions were 65°C for 45 min. For testing, 1 μL of the amplified product was diluted with 100 μL of LFIA running buffer and loaded onto a dual-color test strip. Two independent operators performed visual interpretation within 5–10 min; discrepancies were resolved by a third reviewer. Diagnostic performance metrics for the LAMP-LFIA assay were calculated, including sensitivity, specificity, and overall agreement rate with the RT-qPCR reference standard.

## 3 Results

### 3.1 Principle of the duplex bicolor LAMP-LFIA assay

**FIGURE 1:**
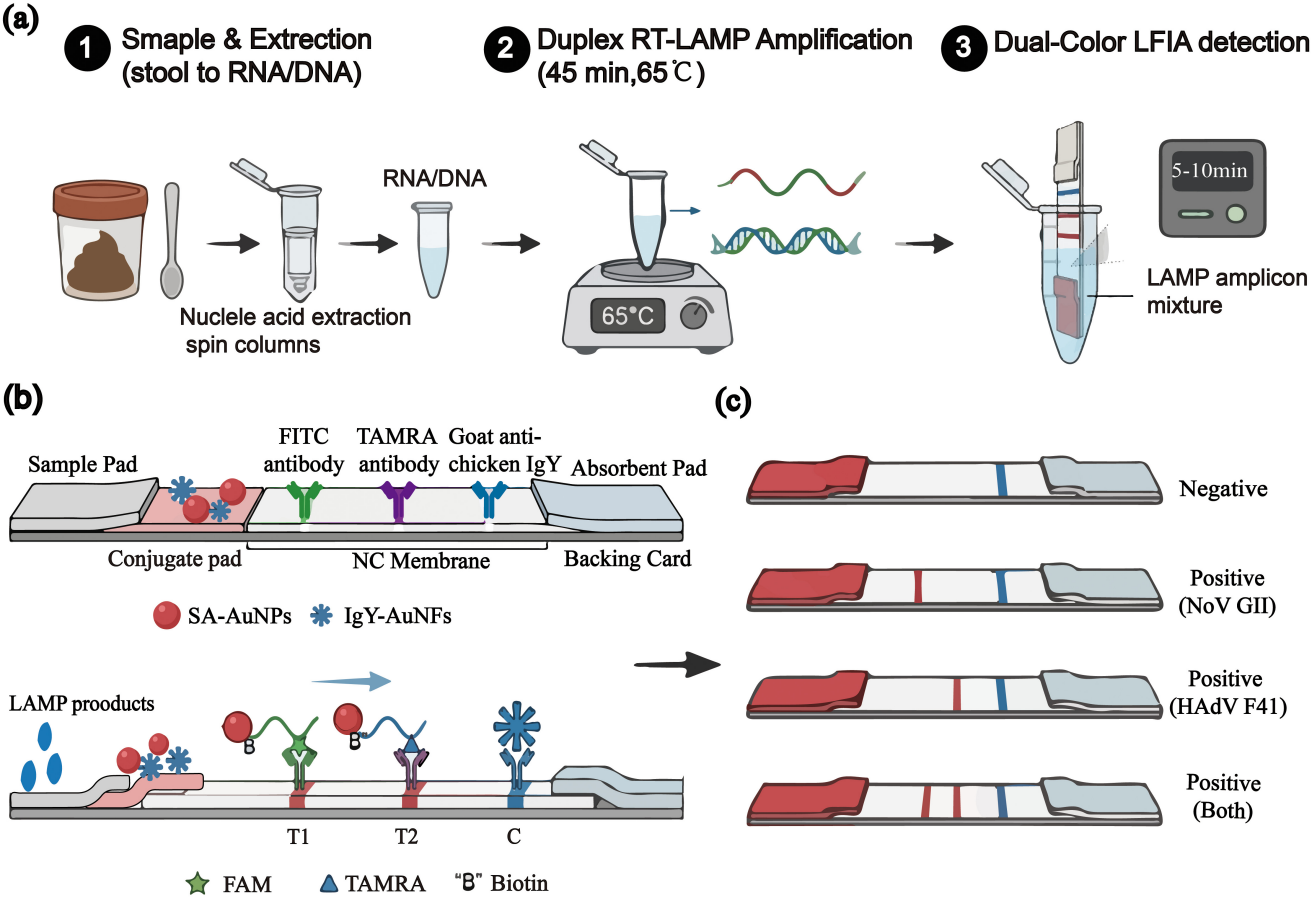
Schematic of the duplex LAMP-LFIA method. **(a)** Workflow from nucleic acid extraction to dual-color lateral flow detection. **(b)** Components of the immunochromatographic strip and mechanism for detecting FAM- and TAMRA-labeled LAMP amplicons. **(c)** Representative readouts for negative, norovirus GII-positive, HAdV F41-positive, and co-infected samples.

The working principle of the developed assay for the simultaneous detection of NoV GII and HAdV-F41 is illustrated in Figure 1. Briefly, viral RNA/DNA is first extracted and subjected to a one-step duplex reverse transcription loop-mediated isothermal amplification (RT-LAMP) at a constant temperature of 65°C. The specially designed inner primers generate biotinylated amplicons that are also labeled with haptens: FAM for NoV GII and TAMRA for HAdV-F41. After amplification, a small aliquot of the product is mixed with running buffer and applied to the sample pad of the LFIA strip. The amplicons migrate by capillary action and first encounter the conjugate pad, where the biotin moieties bind to the streptavidin-conjugated gold nanoparticles (SA-AuNPs), forming colored complexes. These complexes continue to flow across the nitrocellulose membrane. The FAM-labeled complexes are specifically captured by the anti-FITC antibodies immobilized at the T1 line, while the TAMRA-labeled complexes are captured by the anti-TAMRA antibodies at the T2 line, both resulting in the accumulation of red AuNPs. Excess IgY conjugated AuNFs from the conjugate pad are captured by the goat anti-chicken IgY at the C line, generating a distinct blue band that confirms the valid operation of the strip. The entire process, from sample to visual result, is completed within 45 min, enabling clear differential diagnosis for epidemiological and antimicrobial stewardship purposes based on the presence or absence of the red T1 and T2 lines alongside the blue C line.

### 3.2 Optimization of the duplex RT-LAMP conditions

To achieve balanced and efficient co-amplification of Norovirus genogroup II (NoV GII) and human adenovirus F41 (HAdV F41), the duplex RT-LAMP conditions were optimized stepwise, considering the primer ratio and reaction temperature.

**FIGURE 2:**
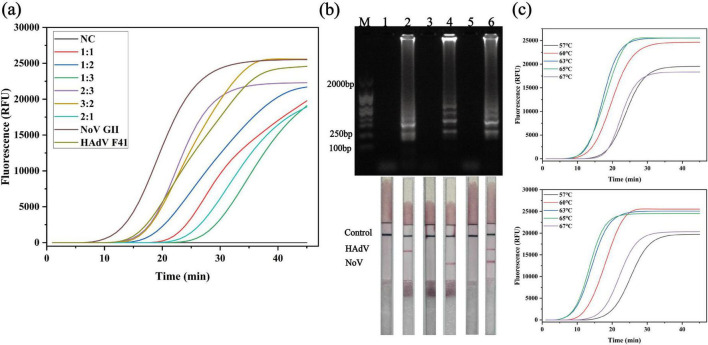
Optimization of duplex RT LAMP conditions for detection of NoV GII and HAdV F41. **(a)** Real-time fluorescence curves of total amplification signals using different NoV: HAdV primer ratios (1:1–2:1), along with the amplification kinetics of monoplex controls (NoV GII and HAdV F41 single primer sets + single templates). **(b)** Agarose gel (top) and corresponding LFIA strips (bottom) of products amplified with the 3:2 primer ratio. Strips: 2, HAdV only; 4, NoV only; 6, dual positive (GII and HAdV F41); 1,3,5, no template controls (triplicate); M, DNA marker. **(c)** Temperature optimization (57–67°C) at the fixed 3:2 primer ratio.

First, the molar ratio of NoV GII to HAdV F41 primer sets was systematically evaluated using several combinations (1:1, 1:2, 1:3, 2:3, 3:2, and 2:1). As shown in Figure 2a, both targets could be amplified under all tested conditions. Still, the amplification kinetics differed substantially among ratios. Compared with other combinations, the 3:2 (NoV: HAdV) primer ratio provided the most synchronized amplification, characterized by similar time-to-threshold (Tt) values for NoV GII and HAdV F41 and robust fluorescence growth without appreciable delay or suppression of either target. In contrast, ratios such as 2:3 tended to favor one target over the other, resulting in unbalanced amplification kinetics. Therefore, the 3:2 ratio was selected as the optimal primer proportion for duplex RT LAMP.

To further verify the performance and specificity of the duplex system under the optimized primer ratio, the corresponding LAMP products were analyzed by agarose gel electrophoresis and LFIA (Figure 2b). Using the 3:2 primer ratio, six reactions were set up: lane 1 contained duplex primers with both NoV GII and HAdV F41 templates (dual positive reaction); lane 3 contained duplex primers with NoV GII template only; lane 5 contained duplex primers with HAdV F41 template only; lanes 2, 4, and 6 contained duplex primers without template (no template controls). Strong, characteristic ladder-like LAMP bands were observed in lanes 1, 3, and 5, confirming efficient amplification in the dual-positive and single-positive reactions. In contrast, no visible bands appeared in lanes 2, 4, and 6, indicating the absence of non-specific amplification. The inclusion of three independent no-template controls (lanes 2, 4, and 6) demonstrates the high robustness of the system against contamination and primer dimer–driven false positives. The LFIA strips corresponding to these reactions showed clear control lines in all tests, while NoV and/or HAdV test lines were only present in the dual-positive and respective single-positive reactions, fully consistent with the gel results, and support the reliability of the visual readout.

With the primer ratio fixed at 3:2, the incubation temperature was then optimized over a range of 57–67°C. As shown in Figure 2c, both NoV GII (upper panel) and HAdV F41 (lower panel) could be amplified across this temperature range, but the amplification efficiency varied. Reactions performed at 65°C consistently yielded the shortest Tt values and the highest endpoint fluorescence intensities for both targets, indicating that 65°C provided the most favorable compromise between reaction speed and amplification efficiency in the duplex format. Consequently, a NoV:HAdV primer ratio of 3:2 combined with an incubation temperature of 65°C was selected as the optimal condition for the duplex RT-LAMP assay.

### 3.3 Synthesis and characterization of gold nanomaterial conjugates

**FIGURE 3:**
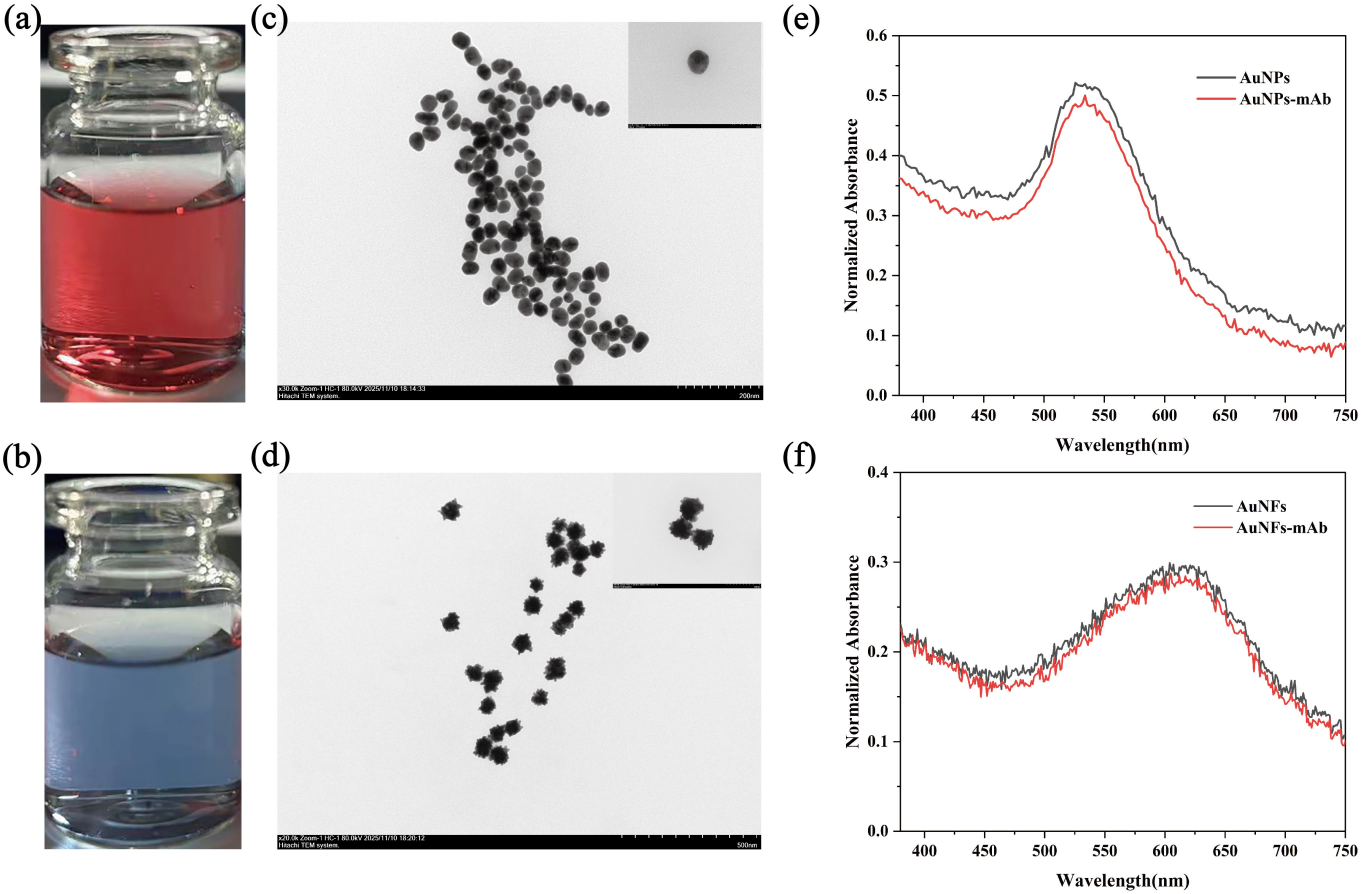
Synthesis and characterization of AuNPs, AuNFs, and their conjugates. **(a)** Photographs of spherical AuNPs (wine-red) and AuNFs (blue) **(b)** dispersions. **(c)** TEM image of monodisperse, ∼40 nm spherical AuNPs (inset: single particle). **(d)** TEM image of branched, ∼80 nm AuNFs (inset: single nanoflower). **(e)** UV–vis spectra of bare and streptavidin-conjugated AuNPs, showing a red shift and broadening upon functionalization. **(f)** UV–vis spectra of bare and antibody-conjugated AuNFs.

Two types of gold nanomaterial conjugates were prepared and employed as colorimetric reporters for the bicolor lateral flow immunoassay. As shown in Figures 3a,b, the colloidal suspension of citrate-reduced spherical (AuNPs) exhibited the characteristic wine-red color associated with well-dispersed AuNPs, whereas the AuNFs displayed a distinct blue hue, allowing straightforward visual discrimination between the two labels. Transmission electron microscopy (TEM) was used to characterize the morphology and size of the synthesized nanomaterials. The AuNPs were nearly spherical, uniformly dispersed, and had an average diameter of approximately 40 nm (Figure 3c), consistent with typical colloidal gold prepared by citrate reduction. AuNFs exhibit a well-defined, branched “flower-like” architecture with an overall size of approximately 80 nm (Figure 3d), reflecting their anisotropic growth. The optical properties of both gold nanomaterials were further confirmed via UV-vis spectroscopy. Spherical AuNPs exhibit a single, narrow surface plasmon resonance (SPR) peak with a main peak around 530 nm (Figure 3e). AuNFs exhibited a broader and more complex absorption spectrum, with a primary peak near 528 nm accompanied by a distinct long-wavelength shoulder extending beyond 600 nm (Figure 3f), consistent with the multiple plasmon modes generated by their branched and anisotropic structure. Biofunctionalization of the nanomaterials was achieved by attaching SA to AuNPs and IgY to AuNFs. Successful conjugation was evidenced by a slight red shift and broadening of the SPR bands for both AuNPs and AuNFs after modification, as compared with their unconjugated counterparts (Figures 3e,f), indicating changes in the local refractive index and surface chemistry upon attachment of the biomolecules.

Taken together, the well-defined spherical and flower-like morphologies, combined with their distinct and tunable optical signatures and stable biofunctionalization, provided a robust material platform for the subsequent development of a duplex lateral flow assay with clear visual color discrimination.

### 3.4 Optimization of the lateral flow immunoassay strip

**FIGURE 4:**
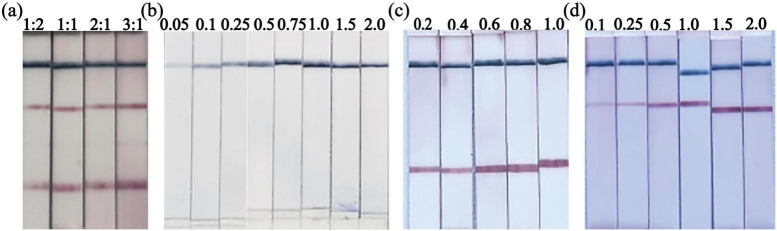
Optimization of the LAMP-LFIA. **(a)** Optimization of Volume Ratio between SA AuNPs and IgY-AuNFs (1:2, 1:1, 2:1, and 3:1 v/v). **(b)** Optimization of C-line goat anti-chicken IgY concentration (0.05, 0.1, 0.25, 0.5, 0.75, 1.0, 1.5, 2 mg/mL). **(c)** Optimization of T1 line anti FITC streaking concentration (0.2, 0.4, 0.6, 0.8, 1.0 mg/mL). **(d)** Optimization of T2 line anti TAMRA streaking concentration (0.1, 0.25, 0.5, 1.0, 1.5, 2.0 mg/mL).

The performance of the duplex LFIA strip was optimized by evaluating the conjugate formulations and the concentrations of immobilized capture reagents. The minimum stabilizing concentration for conjugating SA to the spherical AuNPs and IgY to the AuNFs was determined to be 1 mg/mL via a NaCl flocculation assay, ensuring the colloidal stability of the resulting SA-AuNP and IgY-AuNFs conjugates. The volumetric mixing ratio of these conjugates on the pad was then evaluated. Testing showed that a 1:1 ratio (SA-AuNPs to IgY-AuNFs, v/v) provided the most balanced and intense colorimetric signals, generating robust red bands at both T-lines and a sharp blue control (C) line with minimal background (Figure 4a). Other test ratios resulted in compromised signal balance or contrast. Additionally, the concentration of labeled antibodies on the nitrocellulose membrane was fine-tuned (Figures 4b–d). A stable and distinct blue band was obtained using 0.75 mg/mL goat anti-chicken IgY on line C. For the test lines, experimental validation determined the optimal concentration of FITC-labeled monoclonal antibody on line T1 to be 0.6 mg/mL (specific for the norovirus GII amplicon) and the optimal concentration of TAMRA-labeled monoclonal antibody on line T2 to be 1.0 mg/mL (specific for the HAdV-F41 amplicon). By adjusting the concentrations of the above reagents, the test strip displayed a clear test line (red) and control line (blue) while maintaining a low background, thereby establishing a sensitive and visually distinct dual-detection system for subsequent validation.

### 3.5 Analytical performance of the duplex LAMP-LFIA assay

**FIGURE 5:**
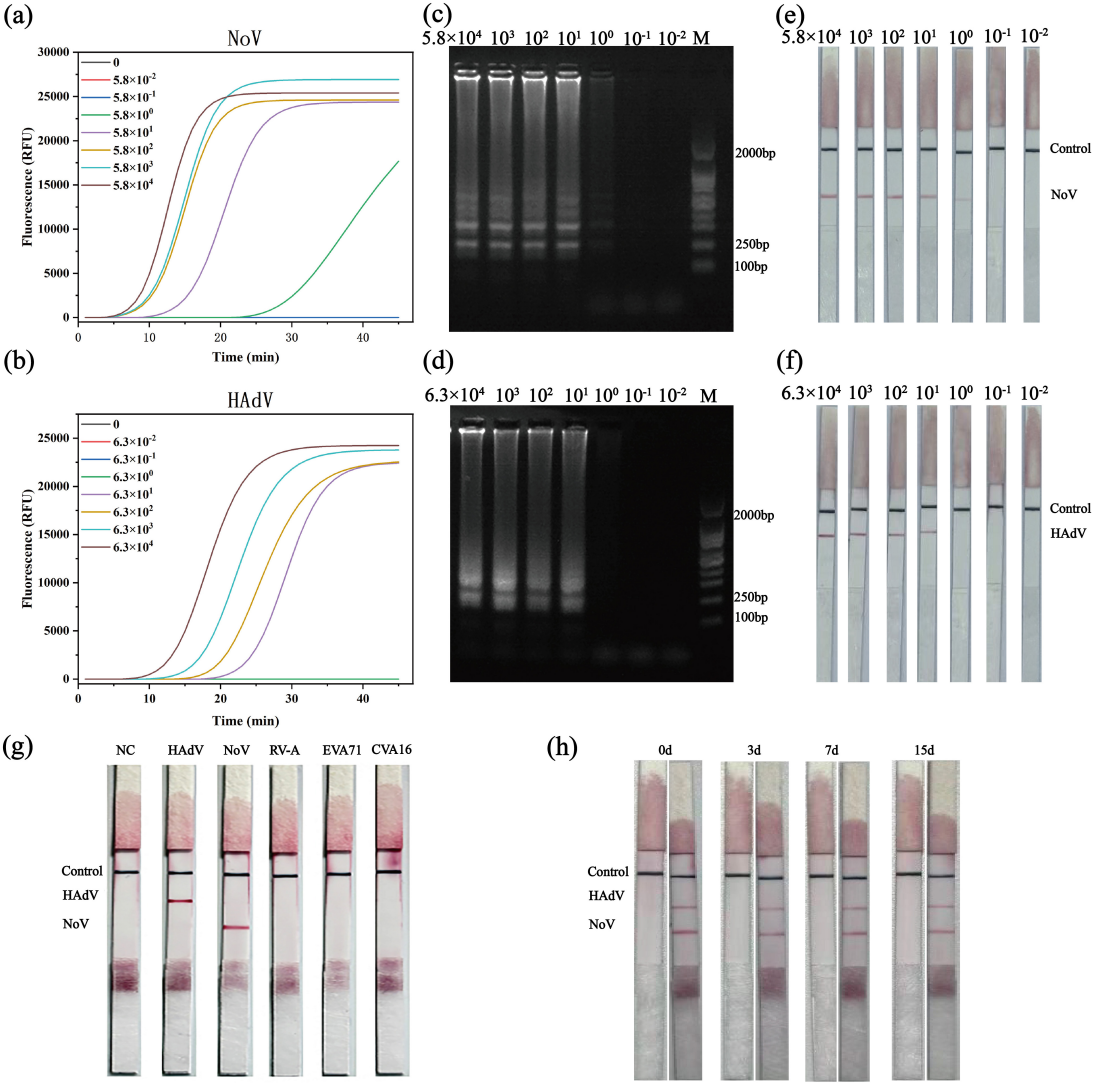
Analytical performance evaluation of the duplex LAMP-LFIA assay. **(a,b)** Real-time fluorescence curves of LAMP using serial dilutions (10^4^–10^–2^ copies/μL) of NoV GII **(a)** and HAdV-F41 **(b)** standards. **(c,d)** Agarose gel analysis of LAMP products from the dilution series in **(a,b)**, showing ladder-like patterns. M, DNA marker. **(e,f)** LFIA readout for NoV GII **(e)** and HAdV-F41 **(f)** from the same dilutions. **(g)** Specificity against non-target enteric viruses (RVA, EVA71, CVA16, ∼10^4^ copies/μL). No cross-reactive signals at T1 (NoV) or T2 (HAdV). NC, no-template control. **(h)** Accelerated stability of LFIA strips stored at 37°C for 0, 3, 7, and 15 days.

The analytical performance of the developed duplex LAMP-LFIA detection method was strictly evaluated in terms of sensitivity, specificity, and storage stability (Figure 5).

#### 3.5.1 Sensitivity and detection limit

##### 3.5.1.1 Sensitivity and limit of detection

The analytical sensitivity of the duplex LAMP-LFIA assay was evaluated using 10-fold serial dilutions of NoV GII RNA and HAdV-F41 DNA standards. The initial concentrations were 5.8 × 10^4^ copies/μL for NoV GII and 6.3 × 10^4^ copies/μL for HAdV-F41, and each was diluted down to 5.8 × 10^–2^ copies/μL and 6.3 × 10^–2^ copies/μL, respectively. Amplification was monitored in real time by fluorescence detection.

As shown in Figures 5a,b, both viruses obtained a typical S-type amplification curve. The positive time is negatively correlated with the initial template concentration, which is in line with the high efficiency of LAMP technology in the wide dynamic range (Nwe et al., 2024). In order to confirm the specificity of the amplification product, especially at low template concentration, the agarose gel electrophoresis analysis of the amplification product of the whole dilution series was carried out. As shown in Figures 5c,d, characteristic ladder-like bands were observed with a clear stepped strip pattern, indicating that the LAMP amplification of NoV GII and HAdV-F41 is specific, and the band intensity weakens with the decrease of template concentration (Fukuda et al., 2006). These results were consistent with the real-time fluorescence monitoring data (Figures 5a,b).

Then, the LAMP amplification product was visually tested with a two-color LFIA test strip. The control line (C line) is blue, the T1 line shows red indicating NoV GII positive, and the T2 line shows red indicating HAdV F41 positive. The template concentration is from high to low, and the test line (T1, T2) shows that the red stripe is also from dark to light until it does not show color. With the decrease of template concentration, the intensity of the red stripe at the test line gradually weakens, which further verifies the quantitative relationship between the initial virus load and the detection signal. The results show that the LOD of NoV GII is 5.8 copies/μL (Figure 5e), and the LOD of HAdV-F41 is 6.3 × 10^1^ copies/μL (Figure 5f).

#### 3.5.2 Analytical specificity

In order to verify the specificity of the method, the nucleic acid standards of non-target pathogens such as rotavirus A (RVA), enterovirus A71 (EVA71), and Coxsackie virus A16 (CVA16) were used, respectively. As shown in Figure 5g, there is no red strip on the test strip test line, and the control line is clearly visible. The positive controls of NoV GII and HAdV F 41 produced strong red stripes on their respective test lines. These results show that the detection method has high analytical specificity and will not cross-react with non-target viruses (Figure 5g).

#### 3.5.3 Storage stability

The stability of LFIA test strips is a key factor in on-site testing applications. We conducted tests using accelerated aging experiments. The test strips were stored at 37°C for 3, 7, and 15 days, then tested with NoV GII and HAdV-F41 nucleic acid standards at concentrations near the limit of detection (approximately 10^1^ copies/μL for NoV GII and 10^2^ copies/μL for HAdV-F41, corresponding to approximately 2 × LOD). As shown in Figure 5h, all aged strips retained performance comparable to that of the fresh ones, with no significant degradation in the signal intensity of positive controls (for both NoV and HAdV) and no emergence of false-positive signals in the negative controls. This indicates excellent short-term thermal stability, suggesting that the strips can maintain their diagnostic performance over time under routine storage conditions, facilitating deployment in resource-limited settings.

### 3.6 Application in real clinical samples

**FIGURE 6:**
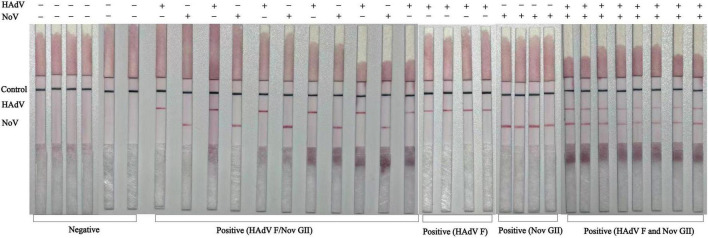
Applicability evaluation of dual LAMP-LFIA detection method in actual positive clinical samples: negative fecal samples (6 cases), NoV positive (9 cases), HAdV positive (10 cases), and both NoV and HAdV positive (8 cases).

To verify the feasibility of the dual color LAMP-LFIA detection method, real positive clinical samples were tested. After initial screening at the Traditional Chinese Medicine Hospital of Xinjiang Production and Construction Corps in Kuitun, China, a total of 33 fecal samples were collected from children with AGE, including 19 positive samples for NoV or HAdV, 8 NoV and HAdV co-infection-positive, and 6 negative samples. The results showed that the negative sample only showed a blue band on the C-line of the test strip. When testing 19 positive samples, the LFIA test strip displayed both the C line and the corresponding T line simultaneously. In addition, when preparing positive samples containing two viruses by mixing two virus samples, the test strip results showed a blue C line and two red T lines (Figure 6). Calculate the diagnostic performance indicators of LAMP-LFIA, including sensitivity, specificity, and overall compliance with RT-qPCR reference standards, as shown in Table 2.

**TABLE 2:** Comparison of clinical sample validation results between LAMP-LFIA and RT-qPCR.

Number of samples	Target	LAMP-LFIA		RT-qPCR		Pos. predictive value (%)	Neg. predictive value (%)	Sensitivity (%)^1^ *	Specificity (%)^1^ *	Accuracy (%)^1^ *
		Pos.	Neg.	Pos.	Neg.					
33	NoV	17	16	17	16	100	100	100	100	100
	HAdV	18	15	18	15					

^1^*Sensitivity = [TP/(TP + FN)]*100, Specificity = [TN/(TN + FP)]*100. Accuracy = [(TP + TN) (TP + TN + FN + FP)]*100. TP, true positive; FP, false positive; FN, false negative; TN, true negative.

## 4 Discussion

In this study, a duplex RT-LAMP assay coupled with dual-color LFIA was developed for the simultaneous detection of NoV GII and HAdV-F41. This multiplex LAMP system integrates two sets of specific primers in a single reaction and can be completed within 45 min at a constant temperature of 65°C. The introduction of FAM/biotin-labeled primers for NoV GII and TAMRA/biotin-labeled primers for HAdV-F41 enabled target-specific amplification and subsequent capture on the LFIA strip. The dual-color gold nanomaterial signaling system, using red AuNPs for test lines and blue AuNFs for the control line, minimized visual misinterpretation and improved user-friendliness. In addition, the incorporation of a dUTP/UDG anti-contamination system effectively eliminated false positive results caused by carryover contamination. The reliability and practicability of the proposed method were validated through testing with real clinical samples, demonstrating 100% concordance with RT-qPCR results. These results indicate that the method is reliable and practical for point-of-care detection of the two major viral pathogens causing acute gastroenteritis.

LAMP is one of several isothermal amplification methods available today (Park, 2022; Yang et al., 2024). Others include recombinase polymerase amplification (RPA), rolling circle amplification (RCA), strand displacement amplification (SDA), helicase-dependent amplification (HDA), cross-priming amplification (CPA), and nucleic acid sequence based amplification (NASBA) (Glökler et al., 2021; Srivastava and Prasad, 2023; Jirakittiwut and Ratthawongjirakul, 2025). RPA operates at lower temperatures (37–42°C) and is very fast (20–30 min), but it requires expensive reagents and thermolabile enzymes, and primer design is more challenging. RCA produces long amplicons and offers single-molecule sensitivity, but it needs circular templates and is time-consuming. HDA mimics natural DNA replication but has lower sensitivity for low copy targets. NASBA is RNA-pecific but involves a complex multi-enzyme workflow.

CRISPR-Cas-based diagnostic systems have gained attention for their high specificity and single-base resolution. These systems combine isothermal amplification with CRISPR-Cas-mediated cleavage, providing programmable specificity (Kostyusheva et al., 2022; Zhou et al., 2025). However, they typically require two-step reactions (amplification followed by Cas detection), increasing operational complexity and cost. The integration of LAMP with CRISPR-Cas systems, while promising for enhanced specificity, often faces temperature incompatibility issues, as LAMP operates at 65°C while most Cas12a nucleases exhibit optimal activity at 37°C. Although thermostable Cas12b variants or phosphorothioate- modified primers can address this limitation, these approaches increase primer design complexity and system optimization requirements (Bagi et al., 2025). Digital LAMP methods, including chip-based and droplet-based approaches, enable absolute quantification without standard curves and offer single-molecule sensitivity. However, these techniques require sophisticated chip fabrication and imaging equipment, with chip costs potentially limiting widespread adoption in resource-limited settings. Our integrated LAMP-LFIA platform combines amplification and detection in a single workflow with visual readout, offering a simpler, more cost-effective alternative for point-of-care applications where absolute quantification is not required. Compared to these techniques, LAMP offers several distinct advantages. It uses 4–6 primers recognizing 6–8 regions of the target, which provides high specificity. The reaction is rapid (usually 30–60 min) and generates large amounts of amplicons (10^8^–10^10^ copies), enabling simple visual detection. LAMP is also highly tolerant to biological inhibitors, allowing direct amplification from minimally processed samples. These features make it well suited for point-of-care testing in resource-limited settings. Nevertheless, LAMP has well-recognized limitations. The use of multiple primers increases the risk of primer-dimer formation and non-specific amplification, leading to false positives. The high concentration of amplification products also makes the method prone to aerosol contamination. In our study, we addressed these issues by optimizing primer design and incorporating a dUTP/UDG anti-contamination system. The 100% specificity observed in clinical samples suggests that these measures are effective.

Despite the promising performance of our assay, several limitations should be acknowledged. While the assay demonstrated excellent specificity against a panel of common non-target enteric viruses, cross-reactivity with other NoV genotypes and other HAdV serotypes was not experimentally evaluated. Although *in silico* analysis suggested minimal homology, experimental validation with a broader panel would further strengthen specificity claims. This is particularly important given the genetic diversity of noroviruses, with multiple genotypes including GII.2, GII.3, GII.4, GII.6, GII.13, and GII.17 coexisting widely (Ai et al., 2025).

Secondly, the assay is currently qualitative, providing visual readout of presence or absence of viral nucleic acids. For applications requiring viral load quantification, qPCR or digital LAMP methods remain the methods of choice. Future iterations could explore integrating smartphone-based image analysis for semi-quantitative interpretation, as demonstrated in recent smartphone-integrated LAMP systems (Yang et al., 2026).

Thirdly, the sample preparation step still requires nucleic acid extraction, which takes approximately 15–20 min and represents a bottleneck for true “sample-to-answer” POCT. However, LAMP’s remarkable tolerance to inhibitors present in complex biological matrices offers potential for direct amplification from minimally processed samples (Natoli et al., 2021; Tripathy et al., 2023). Ongoing work is focused on developing simplified extraction methods, such as thermal incubation, alkaline lysis, or protease-based treatments, that could enable direct detection from raw stool samples.

Finally, the long-term stability of the LFIA strips under various storage conditions requires further investigation beyond the 15-day accelerated aging study presented here (Kaur et al., 2021). Lyophilization or air-drying technologies with appropriate stabilizers could enable long-term room-temperature storage for simplified transportation and field deployment in resource-limited settings (Prakashan et al., 2023).

In summary, the duplex LAMP-LFIA assay developed in this study combines the high sensitivity and specificity of LAMP with the simplicity and visual readout of LFIA. The dual-color detection system minimizes misinterpretation, while the integrated anti-contamination strategies enhance reliability. With its rapid turnaround time, minimal equipment requirements, and excellent performance in clinical sample validation, this platform represents a promising tool for epidemiological surveillance and antimicrobial stewardship programs in resource-limited settings. Future work will focus on expanding specificity validation, simplifying sample preparation, and evaluating long-term stability under field conditions.

## Data Availability

The raw data supporting the conclusions of this article will be made available by the authors, without undue reservation.
