# Supplementary material for: A red/blue bicolor lateral flow immunoassay for simultaneous detection of two enteroviruses based on duplex RT-LAMP
Source: Front Microbiol. 2026 Mar 18;17:1787049. doi: 10.3389/fmicb.2026.1787049 (PMC13038947; doi:10.3389/fmicb.2026.1787049)
Supplement: Supplementary file 1 [file Data_Sheet_1.pdf]

Synthesis of Gold Nanomaterial-Labeled Conjugates

Synthesis of Colloidal AuNPs and Conjugation with Streptavidin

Red colloidal AuNPs with an average diameter of approximately 40 nm were synthesized as the first colorimetric label via the classical citrate reduction method (Memon et al., 2022). Briefly, 49.5 mL of ultrapure water was heated to boiling under vigorous stirring. Subsequently, 0.5 mL of 1% (w/v) trisodium citrate and 0.5 mL of 1% (w/v) HAuCl4 solution were added. The solution's color changed from pale yellow to a wine-red, indicating the formation of nanoparticles. Heating was continued for 5 min. The synthesized AuNPs were cooled to room temperature and stored at 4 °C. The size and morphology were characterized using transmission electron microscopy (TEM), and the absorption spectrum was recorded using a UV-vis spectrophotometer. For conjugation, 5 mL of the AuNPs solution was adjusted to pH 8.0 using 0.2 M K_2CO_3. Then, 70 µL of streptavidin (SA) solution (1 mg/mL) was added drop-wise under gentle stirring. The mixture was incubated overnight at 4°C to allow for adsorption.

Synthesis of AuNFs and Conjugation with IgY

AuNFs were synthesized using HEPES as a reducing agent and particle stabilizer, serving as a second chromogenic label (Fang et al., 2021). Briefly, 30 mL of ultrapure water was stirred under constant conditions, followed by sequential addition of 25 mM HEPES buffer, 1 M sodium hydroxide (NaOH), and 200 µL of 1% (w/v) $HAuCl_4$ solution. The mixture was left to stand at room temperature in the dark for 1 hour to initiate nucleation and growth. Incubate overnight to promote the formation of characteristic branched floral nanostructures. Store at 4°C. AuNFs were characterized using transmission electron microscopy (TEM) and ultraviolet-visible spectroscopy (UV-vis). The unique morphology and plasmonic properties of AuNFs typically exhibit broad absorption peaks at longer wavelengths compared to spherical AuNPs. For the conjugation of AuNFs and IgY, specifically, 5 mL of a freshly synthesized AuNFs solution was adjusted to pH 7.5 using 0.2 M K_2CO_3 solution. Subsequently, 50 µL of IgY solution (1 mg/mL) was slowly added dropwise under stirring. The mixture was left to stand overnight at 4°C to form the IgY-AuNFs complex.

Blocking and Purification of Conjugates

Add the SA-AuNPs and IgY-AuNFs separately to 0.5 mL of 10% (w/v) bovine serum albumin (BSA) solution. Incubate at room temperature for 1 hour to block unbound sites on SA-AuNPs and IgY-AuNFs. Then, centrifuge at 4°C and 10,000g for 30 minutes. Discard the supernatant, and resuspend the pellet in 0.25 mL of suspension buffer. Store at 4°C.
